# Linking Leaf Functional Traits with Soil and Climate Factors in Forest Ecosystems in China

**DOI:** 10.3390/plants11243545

**Published:** 2022-12-15

**Authors:** Xingyu Zhou, Jiaxun Xin, Xiaofei Huang, Haowen Li, Fei Li, Wenchen Song

**Affiliations:** 1 College of Nuclear Technology and Automation Engineering, Chengdu University of Technology, Chengdu 610059, China; 2 College of Life and Environmental Sciences, Minzu University of China, No. 27 Zhongguancun South Street, Haidian, Beijing 100081, China; 3 Applied Nuclear Techniques in Geosciences Key Laboratory of Sichuan, Chengdu University of Technology, Chengdu 610059, China

**Keywords:** leaf functional traits, climate, soil, geographical variation, δ^13^C, δ^15^N, forest ecosystem

## Abstract

Plant leaf functional traits can reflect the adaptive strategies of plants to environmental changes. Exploring the patterns and causes of geographic variation in leaf functional traits is pivotal for improving ecological theory at the macroscopic scale. In order to explore the geographical variation and the dominant factors of leaf functional traits in the forest ecosystems of China, we measured 15 environmental factors on 16 leaf functional traits in 33 forest reserves in China. The results showed leaf area (LA), carbon-to-nitrogen ratio (C/N), carbon-to-phosphorus ratio (C/P), nitrogen-to-phosphorus ratio (N/P), phosphorus mass per area (Pa) and nitrogen isotope abundance (δ^15^N)) were correlated with latitude significantly. LA, Pa and δ^15^N were also correlated with longitude significantly. The leaf functional traits in southern China were predominantly affected by climatic factors, whereas those in northern China were mainly influenced by soil factors. Mean annual temperature (MAT), mean annual precipitation (MAP) and mean annual humidity (MAH) were shown to be the important climate factors, whereas available calcium (ACa), available potassium (AK), and available magnesium (AMg) were shown to be the important climate factors that affect the leaf functional traits of the forests in China. Our study fills the gap in the study of drivers and large-scale geographical variability of leaf functional traits, and our results elucidate the operational mechanisms of forest–soil–climate systems. We provide reliable support for modeling global forest dynamics.

## 1. Introduction

Leaf functional traits can not only reflect plant growth, metabolism and reproduction [1] but also represent plant adaptation strategies to different ecological environments [2,3]. In recent decades, patterns of geographic variation in leaf functional traits at large spatial scales have been paid much attention [4,5,6]. Understanding the geographical variation in leaf functional traits and its relationship with environmental factors can improve the predictions of vegetation changes [7,8,9,10], the large-scale mapping of plant function types [11], analysis of the community structure [12] and dynamic modeling of global vegetation [13,14]. At present, the mainstream view is that the variation in leaf functional traits is mainly affected by climate factors at large scale [15,16,17,18,19,20,21]. Temperature and precipitation were shown to be the most important climate factors, and they have been well known as the dominant factors that affect leaf functional traits, such as leaf area (LA), special leaf area (SLA), nitrogen mass (Nm), leaf phosphorus mass (Pm) and nitrogen-to-phosphorus ratio (N/P) [16,22,23,24]. Humidity is also one of the climate factors affecting leaf functional traits (e.g., Nm, N/P) [25,26]. Furthermore, evapotranspiration has been found to explain 30% of the variation in leaf area [27], and it is one of the limiting factors for some leaf functional traits (e.g., leaf dry matter content (LDMC), Pm, SLA, Nm) [28].

In recent years, soil factors have been shown to be one of the main factors affecting the functional traits at large scale [4,17,18,19,29,30], because the plant–soil interactions are typically the major determinants of changes in processes and functions in forest ecosystems [31]. Leaf functional traits, such as leaf mass per area (LMA), Nm and Pm, are strongly correlated with soil factors, such as available phosphorus (AP), available potassium (AK) available nitrogen (AN) and pH [16,32,33,34]. The carbon-to-nitrogen ratio of leaves (C/N) is associated with the soil salinity [35]. Besides, total nitrogen content (TN) is the main factor affecting LA and SLA, while the total organic carbon (TOC) in soil mainly affects the Pm and Nm of leaves [36]. Soil microorganisms play an important ecological role in soil formation and the protection and regulation of nutrient cycling [37], further changing the physiological adaptation of plants and making soil factors affect leaf functional traits in forest ecosystems [38].

The large latitudinal span of forest ecosystems in China covers all four major forest ecosystem types from north to south, namely, boreal coniferous forests, temperate deciduous broadleaf forests, subtropical evergreen broadleaf forests and tropical forests, making it a valuable sample place for studying geographic variation in leaf functional traits at large scale. Therefore, the variation patterns of leaf functional traits along the gradients of environmental factors (e.g., temperature, precipitation, soil elements and topography) have been widely studied in several climatic regions of China, such as the Tibetan Plateau region [39], the Sanjiangyuan region of northeast China [40], the Karst region of southwest China [35,41], the arid and semi-arid region of northwest China [15,23,42], the deciduous broadleaf forest region of southeastern China [15,43] and the Loess Plateau region of central China [36]. However, at the national scale, only a few reports have explored geographical variation, and most of these studies focused on several species [16,36,44]. Herein, an important question was asked: What are the main factors affecting leaf functional traits in China? The answer to this question will contribute to the global understanding of leaf functional trait variation.

To address this question, we explored the following two hypotheses:

(a) Forest leaf functional traits are mainly correlated with geographic change in climate factors [45];
(b) The geographic change of forest leaf functional traits is mainly influenced by shifts in soil factors [46];

In this study, we sampled soils and leaves from 33 forest reserves in China to test which of the two hypotheses are more realistic. The aim of this study is to link leaf functional traits with soil and climate factors in forest ecosystems in China.

## 2. Results

### 2.1. Geographical Variation

**Figure 1:**
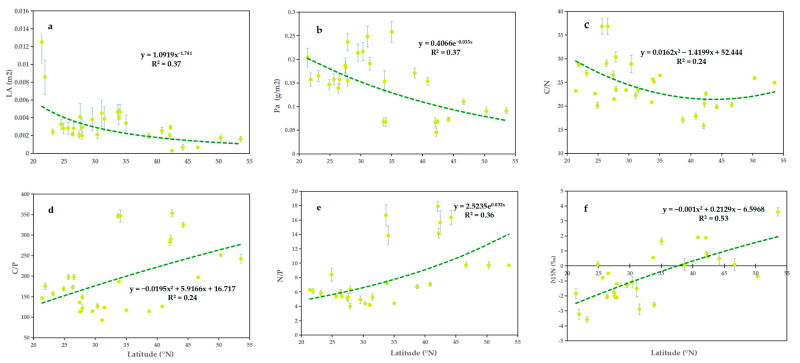
Relationships between latitude and (**a**) LA, (**b**) Pa, (**c**) C/N, (**d**) C/P, (**e**) N/P and (**f**) δ^15^N, respectively.

The leaf area (LA) and phosphorus mass per area (Pa) decreased with increasing latitude (Figure 1a,b); however, the respective curves did not show a good fit (R^2^ = 0.37, *p* < 0.01; R^2^ = 0.37, *p* < 0.01, respectively). The carbon-to-phosphorus ratio (C/P), nitrogen-to-phosphorus ratio (N/P) and nitrogen isotope abundance (δ^15^N) increased with increasing latitude (Figure 1d–f). δ^15^N showed the strongest correlation with latitude (R^2^ = 0.53, *p* < 0.01), whereas those of C/P and N/P were weak, albeit significant (R^2^ = 0.24, *p* < 0.01; R^2^ = 0.36, *p* < 0.01, respectively). In the range of 20–40° N, carbon-to-nitrogen ratio (C/N) decreased with increasing latitude, and in the range of 40–55° N, C/N increased slowly with increasing latitude (R^2^ = 0.24, *p* < 0.05) (Figure 1c).

**Figure 2:**
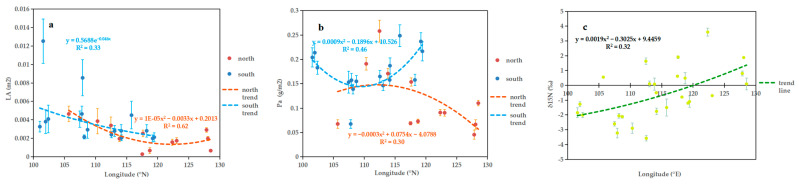
Relationships between longitude and (**a**) LA, (**b**) Pa and (**c**) δ^15^N, respectively.

The LA of leaves from the south was, on average, higher than that of leaves in the north (Figure 2a), which was consistent with the conclusions drawn in Figure 2a. The LA of leaves from the south had a significant but weak negative correlation with longitude (R^2^ = 0.33, *p* < 0.01). Leaves from the north showed a significant and stronger correlation with longitude (R^2^ = 0.62, *p* < 0.01). Between 105° E and 120° E, LA was negatively correlated with longitude, and between 120° E and 130° E, LA was positively correlated with longitude. The phosphorus mass per area (Pa) of southern leaves was generally higher than that of leaves in the north, which is consistent with the conclusion obtained from Figure 1b (Figure 2b). With increasing longitude, the Pa of the southern leaves first decreased and then increased, with the lowest value at 110° E (R^2^ = 0.46, *p* < 0.05). The Pa of the northern leaves increased with longitude and then decreased, with the highest value at 112 °E (R^2^ = 0.30, *p* < 0.05). The degree of variation of δ^15^N with longitude was not significant in the south and the north, but as a whole, δ^15^N showed a significant positive correlation with longitude (R^2^ = 0.32, *p* < 0.01) (Figure 2c).

### 2.2. Environmental Factors Affecting Functional Traits of Leaves

**Figure 3:**
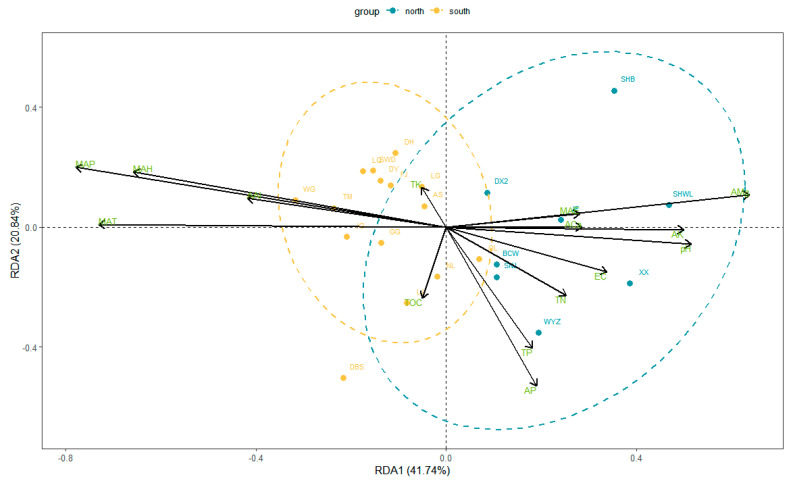
RDA between leaf functional traits and environmental factors.

The RDA showed that the first axis explained 41.74% of the relationship of leaf functional traits and environmental factors, and the second axis explained 20.84%. The mean annual precipitation (MAP), mean annual temperature (MAT), mean annual humidity (MAH), available magnesium (AMg), available potassium (AK) and pH were more strongly correlated with leaf functional traits, followed by correlations with available aluminum (AAl), available phosphorus (AP) and electrical conductivity (EC). AMg, AK, pH, EC and AP, all of which were positively correlated with the first axis, and AMg, AK and pH which were closely associated with the first axis. MAP, MAH, MAT and AAl were negatively correlated with the first axis, with MAT being the most closely related to the first axis. Total potassium (TK) was closely and positively correlated with the second axis, and total organic carbon (TOC) and AP were closely and negatively correlated with the second axis (Figure 3).

We found that the factors influencing the functional traits of southern leaves differed significantly from those of northern leaves (Figure 3). Southern leaf functional traits were mainly influenced by climate factors (MAP, MAH, MAT) and to a lesser extent by soil factors (TK, AAl, TOC). Northern leaf functional traits were mainly influenced by soil factors (AP, TP [total phosphorus], TN [total nitrogen], EC, pH, AK, ACa, AMg) and to a lesser extent by climate factors (MAE). The area of the ellipse where the functional traits of southern leaves were located was significantly smaller than that of the ellipse where the functional traits of the northern leaves were located, thus it can be assumed that the intra-group variability among the functional traits of the southern leaves is smaller than that of the northern leaves.

**Figure 4:**
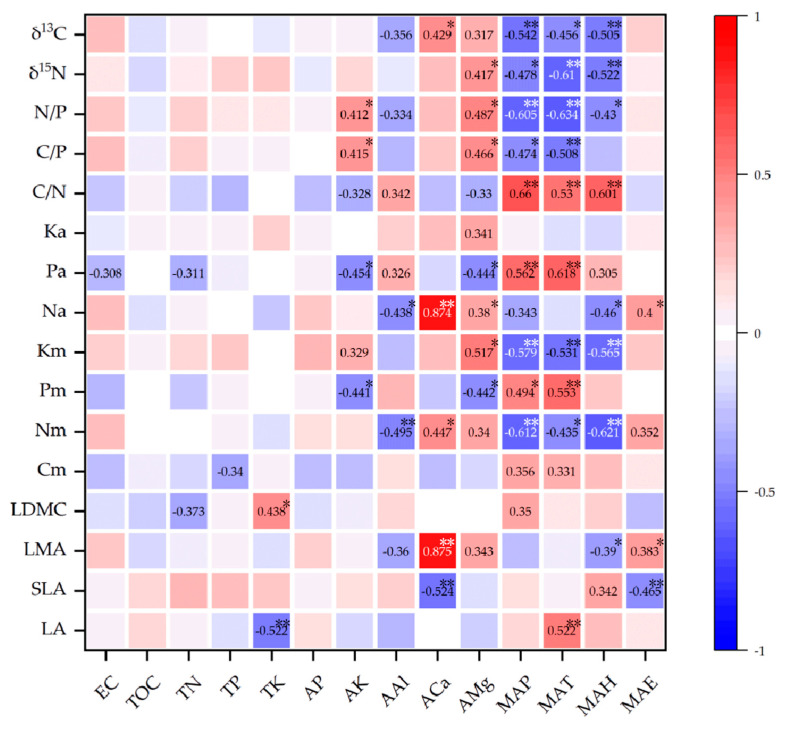
Heatmap of Pearson’s correlations between environmental variables and leaf functional traits. Significant difference was indicated as follows: * < 0.05; ** < 0.01.

We performed pairwise Pearson’s correlation analyses between 16 leaf functional trait variables and 14 environmental variables and produced heat maps (Figure 4). A total of 23 relationship pairs reached significance at the *p* < 0.01 level, and 25 relationship pairs reached significance at the *p* < 0.05 level. Horizontally, all 14 leaf functional traits, apart from potassium mass per area (Ka) and carbon mass (Cm), were significantly influenced by some of the many environmental variables, to varying degrees. Longitudinally, the remaining nine environmental variables, except for EC, TOC, TN, TP and AP, were significantly correlated with certain leaf trait indicators. Among all environmental variables, AMg, MAP, MAT and MAH had the most significant effects on leaf functional traits, and they were significantly correlated with 7, 9, 10 and 8 leaf functional trait variables, respectively. According to the division criteria of a previous study [47], among all significant correlations, only ACa showed strong positive correlations with leaf mass per area (LMA) and nitrogen mass per area (Na), and most of the remaining correlations were moderately correlated in degree, with R^2^ values generally ranging from 0.4 to 0.7, and a small number of correlations were weak.

## 3. Discussion

### 3.1. Geographical Variation in Leaf Functional Traits

The relationship between leaf physiological traits and latitude was not linear in any of our analyses (Figure 1), and this finding was consistent with those of a previous study [20]. The possible reason is that the presence of local habitat heterogeneity reduces the climate variation along the latitudinal gradient [4]. Leaf area (LA) decreased with latitude (Figure 1a), which was in line with the findings of a previous study [48]. Latitude did not have a significant relationship with specific leaf area (SLA) or leaf dry matter content (LDMC), in contrast to previous observations [20,48]. A significant positive correlation between the nitrogen-to-phosphorus ratio (N/P) and latitude was observed (Figure 1e), which was consistent with the observations of one previous study [38] but not with global patterns [45]. The law of N/P change with latitude suggests that, in China, nitrogen is restricted by low latitudes and mainly by phosphorus in high latitudes [49]. The reason for this pattern may be that the dependence of ectomycorrhizal fungi (EMF) on trees is higher at high latitudes than at low latitudes [38]. EMF can inhibit the mycorrhizal root colonization of neighboring arbuscular mycorrhizal herbs by promoting litter accumulation and limiting nutrient access [50,51], and they help trees absorb more N while enhancing their competitiveness, thus leading to higher N/P with increasing latitude [52,53,54,55]. From a general perspective, δ^13^C showed no significant correlation with latitude, although fitting curves differed between north and south [56]. Leaf δ^15^N increased with increasing latitude (Figure 1f), probably because EMF colonization is also positively correlated with latitude [56]. EMF can supply relatively ^15^N-enriched N to their hosts in the rhizosphere [57,58,59]; however, this was contrary to the global trend [46], and it also differed from the trend of increasing and then decreasing with latitude in the southern hemisphere [60]. 

Overall, most leaf functional traits showed no significant longitudinal trends and varied at random, as observed previously [61]. This may be due to the low number of our sampling sites and the lack of a significant trend in hydrothermal conditions between sample sites in the longitude direction [62]. The LA of leaves in southern China decreased significantly with increasing longitude (Figure 2a), which was also found in a study on southern *Taxus mairei* [61]. However, this trend cannot be explained exclusively by precipitation patterns [63]. In contrast, the LA of northern leaves first increased and then decreased with longitude ascending height (Figure 2a). A possible reason for this trend is that, over the longitude of northern sampling sites, the latitude first decreases and then increases, roughly corresponding to decreasing and then increasing warmth. From an overall perspective, the correlation between δ^13^C and longitude is not significant, which is consistent with the conclusions of previous studies [56,64]. δ^15^N showed a significant positive correlation with longitude (Figure 2c). A different study also found that δ^15^N was positively associated with longitude, but the correlation was not significant [56].

### 3.2. Factors Influencing Leaf Functional Traits

#### 3.2.1. Climate Factors

**Figure 5:**
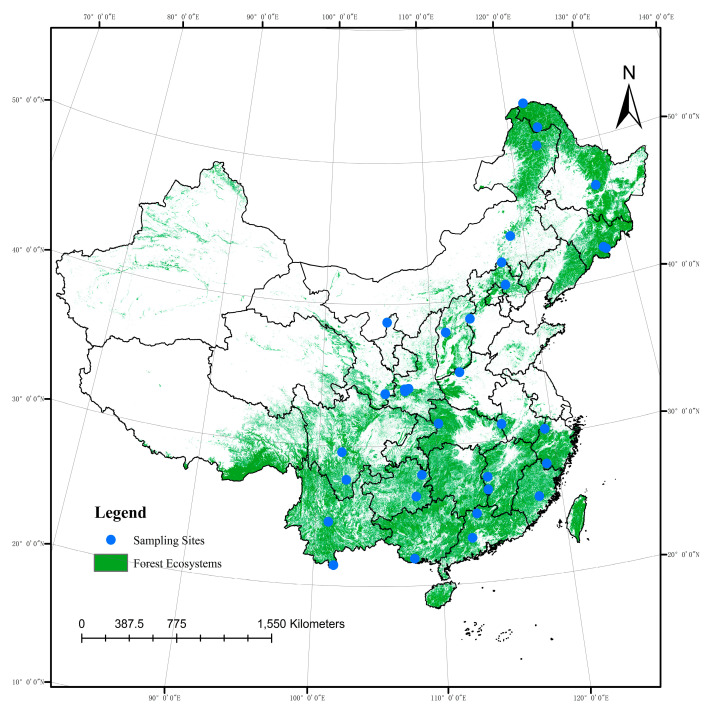
Geographic distribution of the sampling sites.

Mean annual temperature (MAT) showed a significant positive correlation with leaf area (LA) and phosphorus mass (Pm) (Figure 4), as observed previously [45,65]. Long-term monitoring results showed that leaf size increases with increasing temperature, which reflects plant adaptation [66]. The increase of MAT is conducive to the litter decomposition and nutrient circulation, increasing the mineralization rate of nitrogen in the soil, which favors the increase of available nitrogen (AN) in the soil and nitrogen mass (Nm) in the leaves [67]. However, a significant negative correlation between MAT and Nm was found in the present study (Figure 4), which probably occurred due to the presence of other soil elements limiting the growth of Nm [67]. Leaf carbon isotope abundance (δ^13^C) is markedly affected by MAT [38,64,68]. Temperature-related variables were found to exert stronger effects on δ^13^C than precipitation-related factors [12,69]. However, the direction and degree of influence of MAT differed between studies [64,70,71,72]. In the present study, MAT had a significant negative correlation with δ^13^C. MAT was previously shown to have a significant positive correlation with carbon mass (Cm) [73]; however, no such correlation was observed in the current study (Figure 5). No correlation between SLA and MAT was found, by contrast to other studies [20,74].

Mean annual precipitation (MAP) had a significant negative correlation with δ^13^C and LMA, as observed previously [64,75,76]. MAP is the strongest predictor of leaf δ^13^C among global climate variables, and it explains approximately half of the global variation in leaf δ^13^C [77]. When the soil water content and air humidity decrease due to insufficient precipitation, plants may reduce stomatal conductance or stomatal density, leading to improved water-use efficiency and positive leaf δ^13^C in plants [24,78,79,80]. Nm also had a significant negative correlation with MAP (Figure 4). This may be because, under water shortage, plants increase the allocation of N to the leaves, increase osmotic pressure in the cells, reduce the consumption of water by operating at lower stomatal conductance and improve water retention [81,82]. Leaf δ^15^N was negatively correlated with MAP (Figure 4), likely because high moisture levels reduce rhizomicrobial activity and the ability of mycorrhiza to obtain nutrition from decomposing soil organic matter [46,83,84]. However, no correlation of MAP and SLA (or LA) was observed, in contrast to previous studies [65,74,85,86]. This may be because MAP values at all sampling sites were high (>299 mm) and thus did not elicit plant stress.

Increasing water-table depth negatively and directly affects SLA [87], and mean annual evapotranspiration (MAE) is negatively correlated with water-table depth at a country-wide scale [88]. Thus, MAE should be positively correlated with SLA. However, in the current study, MAE and SLA were negatively correlated (Figure 4), which is contrary to the prediction. This discrepancy occurs may be due to the different scales of regional studies [89]. MAH was significantly negatively correlated with δ^13^C (Figure 4), and Liu et al. obtained similar results using *Quercus variabilis* [75].

#### 3.2.2. Soil Mineral Elements

AK had a significant positive effect on N/P (Figure 4), possibly because EMF colonization increases with increasing latitude [55,56], promoting tree roots to deposit their exudates into the soil to facilitate mineral transformation [90,91], thereby increasing AK content [92,93,94]. Higher AK concentrations are beneficial to soil EMF diversity [24,95,96], and EMF are considered to have a facilitating effect on N/P [55]. Nm was significantly positively influenced by ACa (Figure 4). Studies conducted in karst areas similarly found that alkaline soils with Ca^2+^ accumulation promote plant Nm [45]. AMg was positively correlated with δ^15^N and ACa with δ^13^C and LMA, which was observed previously.

## 4. Conclusions

This study found that both climate factors and soil factors significantly affected the leaf functional traits of forests in China; these influences have obvious geographic variability. The leaf functional traits in southern China were predominantly affected by climate factors, whereas those in northern China were mainly influenced by soil factors. Mean annual precipitation (MAP), mean annual temperature (MAT) and mean annual humidity (MAH) were the major climate factors affected leaf functional traits, available magnesium (AMg), available potassium (AK) and available calcium (ACa) were the major soil factors. In this study, it is believed that climatic and geological variation processes dominate the geographical pattern of forest functional traits in China, and their impacts must be comprehensively considered in future studies.

## 5. Materials and Methods

### 5.1. Soil and Leaf Sampling

Leaf and soil samples were collected from 33 mountain forest reserves in China (Figure 1). These forest reserves are located in a latitude range of 21.40°–53.56° N and a longitude range of 101.03°–128.52° E. These areas were characterized by rich vegetation communities (tropical forest, subtropical forest, temperate deciduous broadleaf forest, temperate mixed coniferous–broadleaf forest and boreal forest), mean annual precipitation of 299–2210, mean annual temperature of −5.5–22.7 °C, mean annual humidity of 46.4%–80% and mean annual evapotranspiration of 604–1276 mm. In each forest reserve, 9–15 sampling plots (10 m × 10 m) were randomly selected along the same aspect of the mountain, and 5 topsoil samples (5 cm depth) were randomly collected in each plot and immediately stored in precooled polyethylene bags, at each plot (see Wang et al. [97] for details). Within each plots, 5–10 major species were selected and 20–50 leaf samples on individuals from 2–5 adult healthy trees of each species were collected (see Song and Zhou [56] for details). All meteorological data in the present study were downloaded from the National Meteorological Science Data Center of China (http://data.cma.cn; accessed on 14 April 2019). 

### 5.2. Soil and Leaf Analysis

Basic information on environmental variables and leaf functional traits is shown in Table 1. Soil pH and EC were measured using specific electrodes (Sartorius PB-10, Göttingen, Germany) and a soil suspension (soil and deionized water at 1:2.5). TOC was measured using a Shimadzu TOC Analyzer (TOC-Vcsh; Kyoto, Japan). The SmartChem Discrete Auto Analyzer (SmartChem 200; WESTCO Scientific Instruments Inc., Connecticut, USA) was used to measure TN and TP. Available Ca, Mg, Al, P and K were extracted using Mehlich-III solution and were measured through inductively coupled plasma optical emission spectrometry (ICP-OES, Optima 2100 DV; Perkin-Elmer, Waltham, MA, USA). TK was also measured by ICP-OES after sample digestion. The elemental content of C, N, P and K in the leaves was measured using TOC-Vcsh, SmartChem 200 and Optima 2100 DV instruments, respectively.

After retrieving the leaf samples from the sample points, we performed the following experimental steps: (a) Samples were sorted and washed in distilled water. (b) Leaf thickness, root length and plant height were measured using a Vernier caliper. (c) The leaf area was calculated through Photoshop pixel analysis. (d) The samples were placed in a constant-temperature drying oven for 8 h at 75 °C. (f) An analytical balance was used to record plant mass.

To measure chemical properties, plants were ground with mortar and were sieved through 200 mesh. The values of C%, N%, δ^13^C and δ^15^N were measured using a Finnigan M.A.T 253 Isotope Ratio Mass Spectrometer and Flash 2000 EA-HT Elemental Analyzer (Thermo Fisher Scientific, Waltham, MA, USA). The measurement precisions for δ^13^C and δ^15^N were < ±0.1‰ and < ±0.2‰, respectively (see Song and Zhou [56] for further details).

The determination of δ^13^C and δ^15^N was based on the international standard Peedee Belemnite (PDB) formation and was calculated according to the following Equations (1) and (2):

(1) δ13C=[(C13/C12)Sample(C13/C12)PDB−1]·1000‰

(2) δ15N=[(N15/N15)Sample(N15/N15)PDB−1]·1000‰

where δ^13^C is the thousand percent deviation of sample ^13^C/^12^C from the standard sample; (^13^C/^12^C)Sample is the ^13^C/^12^C of the leaf sample, and (^13^C/^12^C)PDB is ^13^C/^12^C in Peedee Belemnite (South Carolina).

### 5.3. Statistical Analyses

We first performed a DCA test on the leaf functional trait data, and the DCA1 value of axis lengths was 0.459, considerably smaller than 3.0, implying that the data distribution was consistent with the linear model. We therefore used an RDA to test correlations of leaf functional trait indices and environmental factors [98,99,100]. The ‘vegan’ package in RStudio (Integrated Development for R, RStudio Inc., Boston, MA, USA) was used to calculate RDA ordinations. The Pearson’s correlation coefficient between leaf functional traits and environmental variables was calculated using IBM SPSS Statistics (IBM Inc., New York, NY, USA), and a respective heatmap was produced using Origin 2022 (OriginLab Inc., Northampton, MA, USA). The distribution map of forest reserves was produced using ArcGIS 10.4 (ESRI Inc., Redlands, CA, USA). Statistical analyses were performed using Excel 2019 (Microsoft Inc., Redmond, WA, USA). Values are presented as means ± standard error of the mean.

## Figures and Tables

**Table 1:** Leaf functional traits and environmental variables.

Category	Abbreviation	Meaning	Unit
Leaf functional traits	LA	leaf area	m^2^
	SLA	specific leaf area	m^2^·kg
	LMA	leaf mass per area	kg·m^−2^
	LDMC	leaf dry matter content	mg·g^−1^
	Cm	carbon mass	g·kg^−1^
	Nm	nitrogen mass	g·kg^−1^
	Pm	phosphorus mass	g·kg^−1^
	Km	potassium mass	g·kg^−1^
	Na	nitrogen mass per area	g·m^−2^
	Pa	phosphorus mass per area	g·m^−2^
	Ka	potassium mass per area	g·m^−2^
	C/N	carbon-to-nitrogen ratio	-
	C/P	carbon-to-phosphorus ratio	-
	N/P	nitrogen-to-phosphorus ratio	-
	δ^15^N	nitrogen isotope abundance	‰
	δ^13^C	carbon isotope abundance	‰
Soil characteristics	pH	potential of hydrogen	-
	EC	electrical conductivity	μS·cm^−2^
	TOC	total organic carbon	ppm
	TN	total nitrogen	ppm
	TP	total phosphorus	ppm
	TK	total potassium	ppm
	AP	available phosphorus	ppm
	AK	available potassium	ppm
	AAl	available aluminum	ppm
	ACa	available calcium	ppm
	AMg	available magnesium	ppm
Climatic variables	MAP	mean annual precipitation	mm
	MAT	mean annual temperature	°C
	MAH	mean annual humidity	%
	MAE	mean annual evapotranspiration	mm

## Data Availability

The data produced in this study are available in Refs. [38,56,97].
